# A first report of a rare *TP53* variant associated with Li-Fraumeni syndrome manifesting as invasive breast cancer and malignant solitary fibrous tumor

**DOI:** 10.1186/s12957-021-02370-8

**Published:** 2021-08-27

**Authors:** Juraj Prejac, Natalija Dedić Plavetić, Kristina Gotovac Jerčić, Fran Borovečki

**Affiliations:** 1 Department of Oncology, University Hospital Centre Zagreb, Kišpatićeva 12, 10000 Zagreb, Croatia; 2 School of Dental Medicine, University of Zagreb, Gundulićeva 5, 10000 Zagreb, Croatia; 3 School of Medicine, University of Zagreb, Šalata 3, 10000 Zagreb, Croatia; 4 Department of Neurology, University Hospital Centre Zagreb, Kišpatićeva 12, 10000 Zagreb, Croatia; 5 Center for Translational and Clinical Research, Department for Functional Genomics, School of Medicine, University Hospital Centre Zagreb, University of Zagreb, Šalata 2, 10000 Zagreb, Croatia

**Keywords:** *TP53*, Li-Fraumeni syndrome, Invasive breast cancer, Malignant solitary fibrous tumor, Splice-site mutation

## Abstract

**Background:**

Li-Fraumeni is a rare autosomal dominant cancer predisposition syndrome. The basis is a germline mutation of *TP53* gene which encodes tumor suppressor protein resulting in early onset of tumors, most often breast cancer, soft tissue sarcomas, brain tumors, adrenocortical carcinomas, and leukemia.

**Case report:**

We present a case of a young woman with a positive family history for cancer diagnosed with malignant solitary fibrous tumor and luminal B-like invasive breast cancer. Breast cancer and sarcomas account for the majority of tumors associated with Li-Fraumeni syndrome, yet solitary fibrous tumor is a rare clinical entity with no established guidelines for treatment. Even though both primary tumors were successfully resected, the sarcoma relapsed in the form of lung metastases. The NGS analysis revealed single nucleotide variant (c.1101-1G>A) in *TP53* gene, affecting the acceptor splice site at intron 10. Until now, only one case of this genetic variant has been documented with conflicting interpretations of pathogenicity.

**Conclusions:**

The knowledge of *TP53* mutation status is essential since the management of these patients requires different approach to avoid excessive toxicity due to the risk of developing secondary malignancy. Using the clinical criteria to screen for affected individuals facilitates appropriate early genetic counseling of patients and their families. Following the American College of Medical Genetics criteria, we believe that the reported single nucleotide variant (c.1101-1G>A) in *TP53* gene should be considered pathogenic.

**Supplementary Information:**

The online version contains supplementary material available at 10.1186/s12957-021-02370-8.

## Background

Li-Fraumeni syndrome (LFS) is an autosomal dominant-inherited cancer predisposition syndrome characterized by high penetrance and early onset of malignancy [1]. The LFS was defined in 1988 by Frederick Li and Joseph Fraumeni from 24 families with inheritance of high susceptibility to malignant tumors before the age of 45 years [1]. Even though any type of neoplasm may occur, pre-menopausal breast cancer (BC) is observed in 79% of females, followed by soft tissue sarcomas (STSs) in 27% of patients, osteosarcomas, central nervous system tumors, and adrenocortical carcinoma [2]. Germline mutation in the *TP53* gene which encodes tumor suppressor protein p53 is the basis of LFS [3, 4]. Over 50% of all tumors exhibit somatic mutation in *TP53* making it the most frequent target for mutation in human cancer [5]. Considering its role in suppression of carcinogenesis, the risk of cancer development in *TP53* mutation carriers was initially calculated to be 50% before the age of 30 [6]. A more recent conservative approach estimates the prevalence of pathogenic and likely pathogenic *TP53* variants to be within the range of 1 carrier in approximately 4500 individuals [7]; and by the age of 18, 41% of patients develop tumor [2].

The most common female cancer in general population is BC but the mutation frequencies are relatively low in different populations. Most common is the hereditary breast and ovarian cancer syndrome caused by germline mutation in *BRCA1/2* genes, accounting for about 5% of BCs [8, 9]. Germline mutations in TP53 causing LFS are very rare and account for less than 1% of BC cases [10, 11]. On the other hand, STSs are less common heterogeneous group of malignant neoplasms derived from cells of mesodermal origin and only the minority of STSs are associated with inherited syndromes such as LFS [12]. Solitary fibrous tumor (SFT) is a rare type of sarcoma of fibroblastic origin comprising a histologic spectrum of rarely metastasizing mesenchymal neoplasms [13]. The majority of SFTs are usually treated by surgery with the 10-year overall survival rate ranging from 54 to 89% after complete resection of localized disease [14]. The choice of therapy does not significantly differ between sporadic and inherited cases, although some specific features including anatomic location or excessive toxicity may be considered [12].

Two sets of clinical criteria are traditionally used to identify individuals at risk who would benefit from *TP53* mutation testing: the Classic criteria proposed in 1988 [1] and the Li-Fraumeni-like syndrome (LFL) criteria with 2 suggested definitions [15, 16]. More recently, Chompret proposed the proband-related criteria independent of family history [17] which were updated in 2015 [2].

In the modern-day era of personalized and precision medicine, there is a growing need for recognition of hereditary predisposition syndromes as it will allow appropriate genetic evaluation and facilitate timely screening, surveillance, and therapy for patients and their relatives. Here, we present the first case of invasive BC and malignant SFT associated with extremely rare genetic variant of *TP53* gene. To this day, there have been two reports of this variant, one classified as uncertain significance, the other as likely pathogenic [18]. Our particular case and its clinical manifestation provide additional arguments for this variant to be considered pathogenic.

## Case presentation

**Fig. 1:**
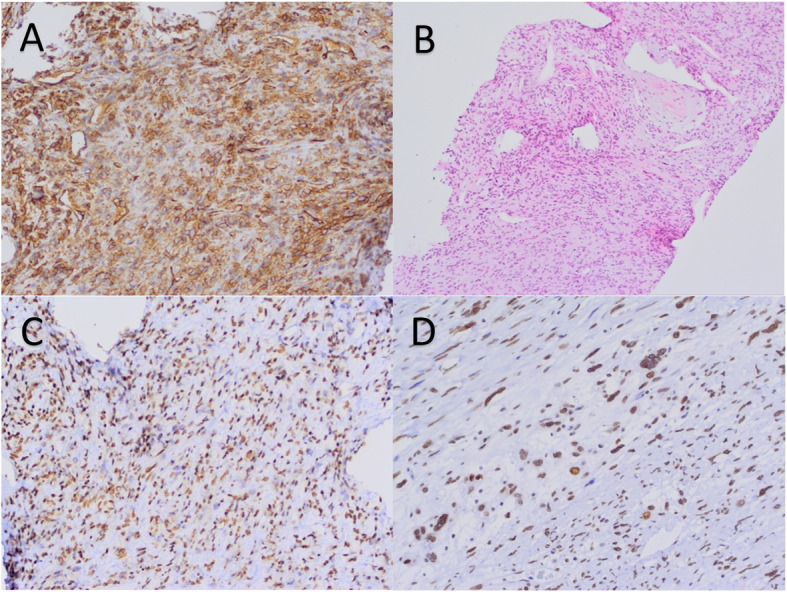
Histopathological analyses of solitary fibrous tumor tissue. **A** Immunohistochemical staining of CD34, typical positive staining on solitary fibrous tumor. **B** Hemalaun-eosin staining of core biopsy tumor tissue from pulmonary metastasis of primary fibrous tumor. **C** Immunohistochemical staining for STAT6 on solitary fibrous tumor (positive nuclear staining, low magnification). **D** Immunohistochemical staining for STAT6 on solitary fibrous tumor (positive nuclear staining, high magnification)

A 36-year-old woman presented with mastalgia and yellowish discharge from her right breast. She reported family history (maternal lineage) positive for cancer. Her mother was diagnosed with BC at 33 and died, and maternal grandmother at 65 years, also from BC. Uncle who had been a smoker died of lung cancer at the age of 70. She has no other live relatives and siblings and has one 3-year-old son. Radiologic imaging found no suspicious lesions in the breast nor enlarged lymph nodes but showed solitary mediastinal mass which was surgically removed. Patohistological examination revealed fibrous tumor with increased mitotic activity, positive for CD34 and STAT6 by immunohistochemistry staining (Fig. 1). Both tumors/breast cancer and solitary fibrous tumor have stained negative for p53 protein.

No further treatment was indicated and after 4 years of follow-up, a lesion in the upper left quadrant of the right breast was discovered. Needle biopsy suggested a luminal B-like invasive BC so she underwent mastectomy and axillary dissection. Surgical pathology specimen showed pT1bN1 grade 3 carcinoma (ER 100%, PR 80%, Her2 negative, Ki 67 26.5%, micrometastasis in 1 out of 11 lymph nodes).

Four cycles of adjuvant treatment with doxorubicin and cyclophosphamide was administered followed by radiotherapy and tamoxifen with ovarian function suppression. The testing for *BRCA1/2* was carried out and found no mutations.

One year later, during preoperative evaluation for breast reconstruction surgery, 4 pulmonary nodular lesions were discovered on chest computed tomography (CT) scan. A CT-guided biopsy of one of the suspected metastases was performed and pathohistological examination was consistent with metastasis of malignant fibrous tumor. Tamoxifen was switched to exemestane due to its possible proliferative effect on fibrous tumor. Chemotherapy for stage IV sarcoma with temozolomide and bevacizumab was initiated given the prior anthracycline chemotherapy.

In the light of the two known malignancies, the patient was referred to genetic counseling and in contrast to prior *BRCA1/2* gene testing only, the multigene panel testing was performed. For the testing purposes, DNA was isolated from peripheral blood using TruSight Rapid Capture commercial reagent kit (Illumina Inc.). DNA sequencing was conducted on a MiSeq sequencing platform (Illumina Inc.) with obtained data analyzed using the Variant Interpreter software (Illumina Inc.) and by searching the OMIM, ClinVar, HGMD, dbSNP, COSMIC, gnomAD, and 1000 Genomes databases. The above methods confirmed a germline single nucleotide variant: (c.1101-1G>A) in *TP53* gene. She was found to be heterozygous for single nucleotide variant (c.1101-1G>A) affecting the acceptor splice site at intron 10 in *TP53* gene, consistent with LFS. This sequence is submitted to ClinVar database, accession number SCV001161439. There were no live siblings for additional germline testing and a 3-year-old son is not permitted for testing without father’s permission according to Croatian laws.

In order to gain additional insight into the genetic and molecular profile of the tumor, a comprehensive genomic profiling (CGP) by a hybrid capture-based DNA sequencing platform was done from tumor tissue obtained from one of the pulmonary metastases. CGP performed by Foundation Medicine, Inc. identified two *TP53* short variant events: Y163N at 19.4% allele frequency and splice site 1101-1G>A at 62.1% allele frequency. The report also revealed a microsatellite stable tumor harboring a relatively low mutational burden of 7 Muts/Mb associated with lower rates of clinical benefit from treatment with PD-1- or PD-L1-targeting immune checkpoint inhibitors [19, 20]. Furthermore, additional mutations in *PTCH1* (subclonal), *PDGFRB*, and the NAB2-STAT6 fusion were confirmed as a somatic event in tumor tissue, none of which have reported therapeutic options with clinical benefit in patient’s tumor type. The list of genes tested has provided in the Supplementary materials.

## Discussion and conclusions

Tumor suppressor protein p53 plays an important role in regulating the cell metabolism and as the key tumor suppressor is mutated in over half of all human cancer [21]. One study suggested *TP53* as independent negative prognostic marker in breast cancer, although this can be debated as the responses to treatment with anthracyclines range from complete remission to progressive disease [22]. According to the International Agency for Research on Cancer Database, 437 variants of *TP53* have been identified to date [23].

Our patient had a relatively high age of onset of first malignancy associated with LFS. Average age for diagnosis of LFS-related cancer in women is 28 years, and even lower when breast cancers in female mutation carriers are excluded [2].

LFS-related BCs are predominantly positive for hormone receptors (84%) and/or HER2/neu (63% of the invasive BCs and 73% of in situ BCs) [3, 24]. Newer study proposes that American College of Medical Genetics and Genomics/Association for Molecular Pathology (ACMG/AMP) *TP53*-specific PP4 criterion includes identification of HER2+ status for BC diagnosed before the age of 40 in addition to PS4 for clinical criteria if the specific clinical criteria are met [25].

Mastectomy is recommended rather than lumpectomy in order to reduce the risks of a second primary BC and avoid radiation due to the possibility of inducing secondary cancer [3, 26]. Considering the increased risk of late toxicity and secondary malignancy from cytotoxic treatments in individuals who carry *TP53* mutation, targeted biological agents seem to be a safer therapeutic option [27]. Furthermore, the patient had adjuvant estrogen receptor modulation therapy replaced with aromatase inhibitor plus ovarial suppression with goserelin as tamoxifen has been associated with increased risk of uterine sarcoma [28].

Next to BC, STSs are the most common malignancy associated with LFS [2], and SFT is by itself a rare tumor of unknown etiology that accounts for 2% of soft tissue tumors [29]. Even though it may be found anywhere in the body, the majority are intra-thoracic with 50-80% being discovered incidentally on chest imaging as asymptomatic masses [29]. Given that SFT is a rare tumor, there are no established guidelines for management and complete surgical resection has remained the mainstay of treatment with low rates of local recurrence as well as progression to metastatic disease [14]. Malignant variant of SFT are, on the other hand, far more likely to recur [29]. Furthermore, inclusion of molecular factors such as *TP53* and TERT promoter status may be prognostic indicators considering tumors with these mutations are classified as high risk and patients almost always develop metastases and die [30]. There is no standard chemotherapy for malignant SFT and the combination of temozolomide and bevacizumab appears the most promising with partial response seen in 79% of cases [14].

When LFS is suspected, different sets of clinical criteria are used and the combination of Chompret’s and Classic achieves highest level of sensitivity [2, 22]. In our case, only 2 out of 3 of the Classic LFS criteria were positive. These include proband diagnosed with sarcoma before the age of 45, first-degree relative with a cancer diagnosis before the age of 45, and another first- or second-degree relative with cancer diagnosis before the age of 45 or sarcoma at any age [1]. However, she did meet the Chompret’s second criterion which refers to proband having multiple primary tumors (except multiple breast tumors), two of which belong to the LFS tumor spectrum, with the initial cancer occurring before the age of 46 years [2, 17]. Hence, the patient was referred to genetic counseling and testing. This is to our knowledge the first report of single nucleotide variant (c.1101-1G>A) in *TP53* gene manifesting as invasive BC and malignant SFT in woman with positive familial history. The detected variant is a rare germline *TP53* variant affecting the acceptor splice site at intron 10 with conflicting interpretations of pathogenicity according to ClinVar and UMD TP53 databases [18, 31, 32]. Prior documented cases in ClinVar include one classified as uncertain significance and the other as likely pathogenic, from 2016 to 2017, respectively [18]. Recognition of this variant as likely pathogenic is based on one affected individual with childhood adrenocortical tumor without relatives with Li-Fraumeni-component tumors [31]. There were no other live relatives or siblings in this family for germline testing for this variant. Her 3-year-old son will be tested for germline variant as soon as his father gives a consent on testing (according to Croatian law genetic testing of minors is allowed with both parents consenting as they are divorced).

Functional loss of tumor suppressor protein encoded by *TP53* is common for more aggressive cancers [33]. And, as stated in the CGP report, two alterations of *TP53* gene are found in tumor, both have been functionally characterized as inactivating and/or result in the disruption or partial or complete loss of the region encoding the TP53 DNA-binding domain (Y163N) or the tetramerization domain (splice site 1101-1G>A) such as observed here, are thought to dysregulate the transactivation of p53-dependent genes and are predicted to promote tumorigenesis [34–36]. According to data on histopathologic features of breast cancer in Li-Fraumeni syndrome published recently, IHC staining of p53 protein depends on type of mutation. High p53 IHC expression was seen in tumors from individuals with germline *TP53* missense mutations, whereas little or no protein expression (< 1% nuclear expression, null pattern) was seen in tumors from carriers of non-missense mutations [37]. In our patient, there were no p53 IHC expressions either on sarcoma tissue or breast carcinoma tissue.

Initially, our patient was tested for *BRCA1/2* mutations with no prior genetic counseling. Indeed, the majority of germline mutations in patients with BC will be in the *BRCA* genes, albeit a small proportion of families may be in the *TP53* or *PTEN* genes in relation to LFS or Cowden’s syndrome, respectively [7, 8]. An earlier diagnosis of LFS would have influenced the treatment if the patient had been referred for counseling at the time of the first tumor occurrence.

In conclusion, this article reports a never before documented clinical manifestation of a rare *TP53* genetic variant in an individual with two primary malignancies. Taking into account the presented information and following ACMG criteria, we believe that this variant should be considered pathogenic. Furthermore, the report highlights the potential benefits of early genetic testing of the populations at risk.

## Supplementary Information

**Additional file 1.** List of genes.

## Data Availability

All the data are available in the patient’s medical record.
