# Supplementary material for: A first report of a rare TP53 variant associated with Li-Fraumeni syndrome manifesting as invasive breast cancer and malignant solitary fibrous tumor
Source: World J Surg Oncol. 2021 Aug 27;19:254. doi: 10.1186/s12957-021-02370-8 (PMC8399826; doi:10.1186/s12957-021-02370-8)
Supplement: Supplementary file 1 — Additional file 1. List of genes. [file 12957_2021_2370_MOESM1_ESM.docx]

List of genes:

1. **DNA GENE LIST: ENTIRE CODING SEQUENCE FOR THE DETECTION OF BASE**

**SUBSTITUTIONS, INSERTION/DELETIONS, AND COPY NUMBER ALTERATIONS**

ABL1 ACTB AKT1 AKT2 AKT3 ALK AMER1 (FAM123B or WTX) APC

APH1A AR ARAF ARFRP1 ARHGAP26 (GRAF) ARID1A ARID2 ASMTL

ASXL1 ATM ATR ATRX AURKA AURKB AXIN1 AXL B2M

BAP1 BARD1 BCL10 BCL11B BCL2 BCL2L2 BCL6 BCL7A BCOR

BCORL1 BIRC3 BLM BRAF BRCA1 BRCA2 BRD4 BRIP1 BRSK1

BTG2 BTK BTLA C11orf30 (EMSY) CAD CALR* CARD11 CBFB CBL

CCND1 CCND2 CCND3 CCNE1 CCT6B CD22 CD274 (PD-L1) CD36 CD58

CD70 CD79A CD79B CDC73 CDH1 CDK12 CDK4 CDK6 CDK8

CDKN1B CDKN2A CDKN2B CDKN2C CEBPA CHD2 CHEK1 CHEK2 CIC

CIITA CKS1B CPS1 CREBBP CRKL CRLF2 CSF1R CSF3R CTCF

CTNNA1 CTNNB1 CUX1 CXCR4 DAXX DDR2 DDX3X DNM2 DNMT3A

DOT1L DTX1 DUSP2 DUSP9 EBF1 ECT2L EED EGFR ELP2

EP300 EPHA3 EPHA5 EPHA7 EPHB1 ERBB2 ERBB3 ERBB4 ERG

ESR1 ETS1 ETV6 EXOSC6 EZH2 FAF1 FAM46C FANCA FANCC

FANCD2 FANCE FANCF FANCG FANCL FAS (TNFRSF6) FBXO11 FBXO31 FBXW7

FGF10 FGF14 FGF19 FGF23 FGF3 FGF4 FGF6 FGFR1 FGFR2

FGFR3 FGFR4 FHIT FLCN FLT1 FLT3 FLT4 FLYWCH1 FOXL2

FOXO1 FOXO3 FOXP1 FRS2 GADD45B GATA1 GATA2 GATA3 GID4 (C17orf39)

GNA11 GNA12 GNA13 GNAQ GNAS GPR124 GRIN2A GSK3B GTSE1

HDAC1 HDAC4 HDAC7 HGF HIST1H1C HIST1H1D HIST1H1E HIST1H2AC HIST1H2AG

HIST1H2AL HIST1H2AM HIST1H2BC HIST1H2BJ HIST1H2BK HIST1H2BO HIST1H3B HNF1A HRAS

HSP90AA1 ICK ID3 IDH1 IDH2 IGF1R IKBKE IKZF1 IKZF2

IKZF3 IL7R INHBA INPP4B INPP5D (SHIP) IRF1 IRF4 IRF8 IRS2

JAK1 JAK2 JAK3 JARID2 JUN KAT6A (MYST3) KDM2B KDM4C KDM5A

KDM5C KDM6A KDR KEAP1 KIT KLHL6 KMT2A (MLL) KMT2C (MLL3) KMT2D (MLL2)

KRAS LEF1 LRP1B LRRK2 MAF MAFB MAGED1 MALT1 MAP2K1

MAP2K2 MAP2K4 MAP3K1 MAP3K14 MAP3K6 MAP3K7 MAPK1 MCL1 MDM2

MDM4 MED12 MEF2B MEF2C MEN1 MET MIB1 MITF MKI67

MLH1 MPL MRE11A MSH2 MSH3 MSH6 MTOR MUTYH MYC

MYCL (MYCL1) MYCN MYD88 MYO18A NCOR2 NCSTN NF1 NF2 NFE2L2

NFKBIA NKX2-1 NOD1 NOTCH1 NOTCH2 NPM1 NRAS NT5C2 NTRK1

NTRK2 NTRK3 NUP93 NUP98 P2RY8 PAG1 PAK3 PALB2 PASK

PAX5 PBRM1 PC PCBP1 PCLO PDCD1 PDCD11 PDCD1LG2 (PD-L2) PDGFRA

PDGFRB PDK1 PHF6 PIK3CA PIK3CG PIK3R1 PIK3R2 PIM1 PLCG2

POT1 PPP2R1A PRDM1 PRKAR1A PRKDC PRSS8 PTCH1 PTEN PTPN11

PTPN2 PTPN6 (SHP-1) PTPRO RAD21 RAD50 RAD51 RAF1 RARA RASGEF1A

RB1 RELN RET RHOA RICTOR RNF43 ROS1 RPTOR RUNX1

S1PR2 SDHA SDHB SDHC SDHD SERP2 SETBP1 SETD2 SF3B1

SGK1 SMAD2 SMAD4 SMARCA1 SMARCA4 SMARCB1 SMC1A SMC3 SMO

SOCS1 SOCS2 SOCS3 SOX10 SOX2 SPEN SPOP SRC SRSF2

STAG2 STAT3 STAT4 STAT5A STAT5B STAT6 STK11 SUFU SUZ12

TAF1 TBL1XR1 TCF3 (E2A) TCL1A (TCL1) TET2 TGFBR2 TLL2 TMEM30A

TMSB4XP8 (TMSL3) TNFAIP3 TNFRSF11A TNFRSF14 TNFRSF17 TOP1 TP53 TP63

TRAF2 TRAF3 TRAF5 TSC1 TSC2 TSHR TUSC3 TYK2 U2AF1

U2AF2 VHL WDR90 WHSC1 (MMSET or NSD2) WISP3 WT1 XBP1 XPO1

YY1AP1 ZMYM3 ZNF217 ZNF24 (ZSCAN3) ZNF703 ZRSR2

1. **DNA GENE LIST: FOR THE DETECTION OF SELECT REARRANGEMENTS**

ALK BCL2 BCL6 BCR BRAF CCND1 CRLF2 EGFR EPOR

ETV1 ETV4 ETV5 ETV6 EWSR1 FGFR2 IGH IGK IGL

JAK1 JAK2 KMT2A (MLL) MYC NTRK1 PDGFRA PDGFRB RAF1 RARA

RET ROS1 TMPRSS2 TRG

1. **RNA GENE LIST: FOR THE DETECTION OF SELECT REARRANGEMENTS**

ABI1 ABL1 ABL2 ACSL6 AFF1 AFF4 ALK ARHGAP26 (GRAF)

ARHGEF12 ARID1A ARNT ASXL1 ATF1 ATG5 ATIC BCL10 BCL11A

BCL11B BCL2 BCL3 BCL6 BCL7A BCL9 BCOR BCR BIRC3

BRAF BTG1 CAMTA1 CARS CBFA2T3 CBFB CBL CCND1 CCND2

CCND3 CD274 (PD-L1) CDK6 CDX2 CHIC2 CHN1 CIC CIITA CLP1

CLTC CLTCL1 CNTRL (CEP110) COL1A1 CREB3L1 CREB3L2 CREBBP CRLF2 CSF1

CTNNB1 DDIT3 DDX10 DDX6 DEK DUSP22 EGFR EIF4A2 ELF4

ELL ELN EML4 EP300 EPOR EPS15 ERBB2 ERG ETS1

ETV1 ETV4 ETV5 ETV6 EWSR1 FCGR2B FCRL4 FEV FGFR1

FGFR1OP FGFR2 FGFR3 FLI1 FNBP1 FOXO1 FOXO3 FOXO4 FOXP1

FSTL3 FUS GAS7 GLI1 GMPS GPHN HERPUD1 HEY1 HIP1

HIST1H4I HLF HMGA1 HMGA2 HOXA11 HOXA13 HOXA3 HOXA9 HOXC11

HOXC13 HOXD11 HOXD13 HSP90AA1 HSP90AB1 IGH IGK IGL IKZF1

IL21R IL3 IRF4 ITK JAK1 JAK2 JAK3 JAZF1 KAT6A (MYST3)

KDSR KIF5B KMT2A (MLL) LASP1 LCP1 LMO1 LMO2 LPP LYL1

MAF MAFB MALT1 MDS2 MECOM MKL1 MLF1 MLLT1 (ENL) MLLT10 (AF10)

MLLT3 MLLT4 (AF6) MLLT6 MN1 MNX1 MSI2 MSN MUC1 MYB

MYC MYH11 MYH9 NACA NBEAP1 (BCL8) NCOA2 NDRG1 NF1 NF2

NFKB2 NIN NOTCH1 NPM1 NR4A3 NSD1 NTRK1 NTRK2 NTRK3

NUMA1 NUP214 NUP98 NUTM2A OMD P2RY8 PAFAH1B2 PAX3 PAX5

PAX7 PBX1 PCM1 PCSK7 PDCD1LG2 (PD-L2) PDE4DIP PDGFB PDGFRA PDGFRB

PER1 PHF1 PICALM PIM1 PLAG1 PML POU2AF1 PPP1CB PRDM1

PRDM16 PRRX1 PSIP1 PTCH1 PTK7 RABEP1 RAF1 RALGDS RAP1GDS1

RARA RBM15 RET RHOH RNF213 ROS1 RPL22 RPN1 RUNX1

RUNX1T1 (ETO) RUNX2 SEC31A SEPT5 SEPT6 SEPT9 SET SH3GL1 SLC1A2

SNX29 (RUNDC2A) SRSF3 SS18 SSX1 SSX2 SSX4 STAT6 STL SYK

TAF15 TAL1 TAL2 TBL1XR1 TCF3 (E2A) TCL1A (TCL1) TEC TET1 TFE3

TFG TFPT TFRC TLX1 TLX3 TMPRSS2 TNFRSF11A TOP1 TP63

TPM3 TPM4 TRIM24 TRIP11 TTL TYK2 USP6 WHSC1 (MMSET or NSD2)

WHSC1L1 YPEL5 ZBTB16 ZMYM2 ZNF384 ZNF521
